# Prevalence, genetic diversity and implications for public health of *Enterocytozoon bieneusi* in various rodents from Hainan Province, China

**DOI:** 10.1186/s13071-020-04314-9

**Published:** 2020-09-02

**Authors:** Wei Zhao, Huanhuan Zhou, Ling Yang, Tianming Ma, Jingguo Zhou, Haiju Liu, Gang Lu, Huicong Huang

**Affiliations:** 1 Department of Parasitology, Wenzhou Medical University, Wenzhou, Zhejiang 325035 China; 2 Key Laboratory of Tropical Translational Medicine of Ministry of Education, Hainan Medical University, Haikou, 571199 China; 3 Department of Pathogenic Biology, Hainan Medical University, Haikou, Hainan China; 4 Hainan Medical University-The University of Hong Kong Joint Laboratory of Tropical Infectious Diseases, Hainan Medical University, Haikou, Hainan China

**Keywords:** *Enterocytozoon bieneusi*, Rodent, Genotype, ITS, Zoonotic

## Abstract

**Background:**

Rodents, globally overpopulated, are an important source for zoonotic disease transmission to humans, including *Enterocytozoon bieneusi* (one of the most prevalent zoonotic pathogens). Here, we studied the prevalence and performed genetic analyses of *E. bieneusi* in rodents from the Hainan Province of China.

**Methods:**

A total of 603 fresh fecal samples were gathered from 369 wild rats, 117 bamboo rats, 93 Asiatic brush-tailed porcupine and 24 red-bellied squirrels. The wild rats were identified to the species level by amplification of a 421-bp region of the *cytb* gene from fecal DNA using PCR. Genotype analysis was performed by amplification of the internal transcribed spacer (ITS) region of rDNA of *E. bieneusi* using PCR.

**Results:**

Seven wild rat species were identified. The average rate of infection with *E. bieneusi* was 15.8% (95/603) with 18.7% (69/369) in wild rats, 11.9% (25/210) in farmed rodents and 4.2% (1/24) in red-bellied squirrels. Sixteen *E. bieneusi* genotypes were identified, including 9 known genotypes (D, Type IV, PigEBITS7, Peru8, Peru11, ESH02, S7, EbpA and CHG5), and 7 novel genotypes (HNR-I to HNR-VII). Genotype D (44.2%, 42/95) predominated, followed by PigEBITS7 (20.0%, 19/95), HNR-VII (15.8%, 15/95), Type IV (5.3%, 5/95), HNR-III (2.1%, 2/95), HNR-VI (2.1%, 2/95) and each of the remaining 10 genotypes (1.1%, 1/95). The phylogenetic analysis of the ITS region of *E. bieneusi* divided the identified genotypes into the following four groups: Group 1 (*n* = 13), Group 2 (*n* = 1), Group 12 (*n* = 1), and the novel Group 13 (*n* = 1).

**Conclusions:**

To our knowledge, this is the first report on the identification of *E. bieneusi* in rodents from Hainan, China. The zoonotic potential of the identified *E. bieneusi* genotypes suggested that the rodents poses a serious threat to the local inhabitants. Thus, measures need to be taken to control the population of wild rats in the areas investigated in this study, along with identification of safe methods for disposal of farmed rodent feces. Additionally, the local people should be made aware of the risk of disease transmission from rodents to humans.
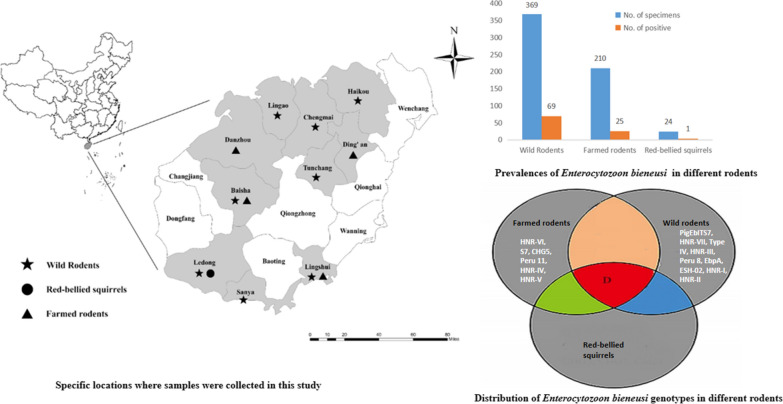

## Background

*Enterocytozoon bieneusi* is a prevalent pathogen in humans and an important zoonotic agent in animals worldwide [1]. The most common clinical symptom of *E. bieneusi* infections is diarrhea for patients with acquired immunodeficiency syndrome (AIDS) or other immunocompromising conditions while asymptomatic infections are common for healthy persons [2]. Humans predominantly acquire infections by ingestion of infectious spores of *E. bieneusi*; potential sources of the spores include water, soil, environmental surfaces, fecal contamination, and improper manure or irrigation water practices in growing fruits and vegetables [3–6]. Thus, understanding the source and mechanism of transmission of *E. bieneusi* could prevent its spread in humans.

Previous studies detected *E. bieneusi* using PCR-based assays with appropriate gene markers [7]. More than 500 genotypes, of which 142 infected humans and 49 infected both humans and animals, were identified through the internal transcribed spacer (ITS) sequence analysis of rDNA of *E. bieneusi* [5, 8, 9]. These genotypes were grouped into 11 genetically isolated clusters by phylogenetic analysis [5]. Group 1 is the largest group containing more than 300 genotypes, including 87.6% (132/142) human-pathogenic genotypes [5, 10]; 85.7% (42/49) zoonotic genotypes also belong to this group [5, 9]. In Group 2, up to 100 genotypes are considered to be adapted to ruminants; however, contrary to previous reports, a broader host range was indicated for some genotypes, such as genotypes I, J, BEB6, BEB4 and CHN3 that are also commonly found in humans, enhancing their importance for public health [5]. Genotypes in Groups 3 to 11 have been mainly reported in specific hosts or wastewater [11]. Group 1 or 2 are frequently reported in pets, non-human primates, wildlife, and livestock (pigs, cattle, sheep, etc.) [5, 8]. However, the mechanism of transmission of infection from a specific animal to humans is unclear.

Rodents have a high rate of multiplication as well as survival rate, which have resulted in their overpopulation. They are also the reservoirs or carriers of several types of zoonotic pathogens, including *E. bieneusi*. Until now, 60 genotypes of *E. bieneusi*, including 18 zoonotic genotypes (C, CZ3, D, BEB6, EbpC, Nig7, Peru6, Type IV, EbpA, PigITS7, H, S7, Peru8, S6, Peru16, J, Peru11 and PigITS5) have been identified in rodents, confirming their role in disease transmission (Table 1) [11–25].

**Table 1:** Prevalence and distribution of *Enterocytozoon bieneusi* genotypes in rodents in the different countries or areas

Country	Rodent species	No. positive/no. examined (%)	Genotype (*n*)	Reference
China	Bamboo rat (*Rhizomys sinensis*)	22/435 (5.1)	**D** (17); **EbpA** (1); **J** (1); **PigEBITS7** (1); BR1 (1); BR2 (1)	12
	Brown rat (*Rattus norvegicus*)	19/242 (7.9)	**D** (17); **Peru6** (2)	13
	Bower’s white-toothed rat (*Berylmys bowersi*)	37/117 (31.6)	**D** (14); **K** (8); **PigEBITS7** (22); **Peru8** (2); CQR-1 (10); CQR-2 (15); CQR-3 (1); GDR-1(2); GDR-2 (1); GDR3 (1)	14
	Edwardʼs long-tailed rat (*Leopoldamys edwardsi*)	39/111 (35.1)		
	Experimental rats^a^	14/291 (4.8)	**EbpA** (7); **EbpC** (3); **CHY1** (2); N (1); SHR1 (1)	15
	Chinchilla (*Chinchilla lanigera*)	5/140 (3.6)	**BEB6** (3); **D** (2)	16
	Chipmunk (*Eutamias asiaticus*)	49/279 (17.6)	SCC-1 (17); SCC-2 (9); **D** (6); **CHY1** (5); SCC-3 (5); **Nig 7** (4); CHG9 (2); SCC-4 (1)	17
	Red-bellied tree squirrel (*Callosciurus erythraeus*)	24/144 (16.7)	**D** (18); **EbpC** (3); SC02 (1); CE01 (1); CE02 (1)	18
	Red squirrel (*Sciurus vulgaris*)	61/314 (19.4)	**D** (27); SCC-2 (18); SCC-3 (12); RS01 (2); RS02 (2)	19
	Rodents^b^	8/199 (4.0)	CHG14 (3); **BEB6** (2); **D** (2); CHG2 (1)	20
Czech-Germany border	East-European house mouse (*Mus m. musculus*)	14/127 (11.0)	**EpbA** (2); **D** (6); **PigEBITS5** (4); **C** (1); **H** (1)	21
	West-European house mouse (*Mus m. domesticus*)	17/162 (10.5)	**CZ3** (4); **PigEBITS5** (3); **D** (4); **Peru 8** (4); **S6** (1); **C** (1)	21
Peru	Guinea pig (*Cavia porcellus*)	7/8 (87.5)	**Preu 16** (7)	22
Poland	Striped field mouse (*Apodemus agrarius*)	79/184 (42.9)	**D** (6); gorilla 1 (1); WR5 (1); WR7 (1); WR8 (2)	23
	Yellow-necked mouse (*Apodemus flavicollis*)	18/60 (30.0)	**D** (2); WR4 (1); WR6 (6); WR1 (1); WR9 (1)	23
	Bank vole (*Myodes glareolus*)	18/46 (39.1)	D (2); WR6 (2); WR10 (2); WR2 (1)	23
	House mouse (*Mus musculus*)	6/21 (28.6)	WR3 (1)	23
Slovakia	House mouse (*Mus musculus musculus*)	3/280 (1.1)	Unknown (3)	24
USA	Eastern gray squirrel (*Sciurus carolinensis*)	11/34 (32.4)	**Type IV** (3); WL4 (5); WW6 (2); PtEbV+WL21 (1)	11
	Eastern chipmunk (*Tamias striatus*)	5/7 (71.4)	**Type IV** (1); WL4 (3); WL23 (1)	11
	Woodchuck (*Marmota monax*)	5/5 (100)	**Type IV**+WL20 (1); WL4 (2); WL22 (1); WW6 (1)	11
	Deer mouse (*Peromyscus* sp.)	13/55 (23.6)	WL4 (10); WL23 (2); WL25 (1)	11
	Boreal red-backed vole (*Myodes gapperi*)	1/5 (20.0)	WL20+WL21(1)	11
	Meadow vole (*Microtus pennsylvanicus*)	3/10 (30.0)	**Peru11** (1); **Peru11+Type IV** (1); WL21+unknown (1)	11
	Prairie dog (*Cynomys ludovicianus*)	14/29 (48.3)	Row (14)	25

^a^Experimental rats including 104 wistar rats, 87 sprague dawley rats and 100 spontaneously hypertensive rats

^b^Rodents including 168 brown rats (*Rattus norvegicus*) and 31 house mice (*Mus musculus*)

*Notes*: Genotypes detected in humans are shown in bold

Previous studies have reported *E. bieneusi* infections in eight rodent species, including, brown rats (*Rattus norvegicus*), bamboo rats (*Rhizomys sinensis*), house mice (*Mus musculus*), Bower’s white-toothed rats (*Berylmys bowersi*), Edward’s long-tailed rats (*Leopoldamys edwardsi*), Chipmunks (*Eutamias asiaticus*), Chinchillas (*Chinchilla lanigera*), and red-bellied squirrels (*Callosciueus erythraeus*) in China. These studies involved five provinces with infection rates ranging from 3.6% to 35.1% [12–20]. In Hainan Province in China there is a high number of rodents that are in close contact with humans and other animals and there is a high prevalence of *E. bieneusi* in farmed, household, and wild animals [8, 9, 26, 27]. Thus, it is necessary to understand *E. bieneusi* epidemiology in rodents to prevent pathogen infection in humans as well as in other animals. This study explored the prevalence of *E. bieneusi* in rodents in several areas of Hainan Province in China, to evaluate the zoonotic potential of the isolates at the genotype level.

## Methods

### Rodent fecal sample collection

**Fig. 1:**
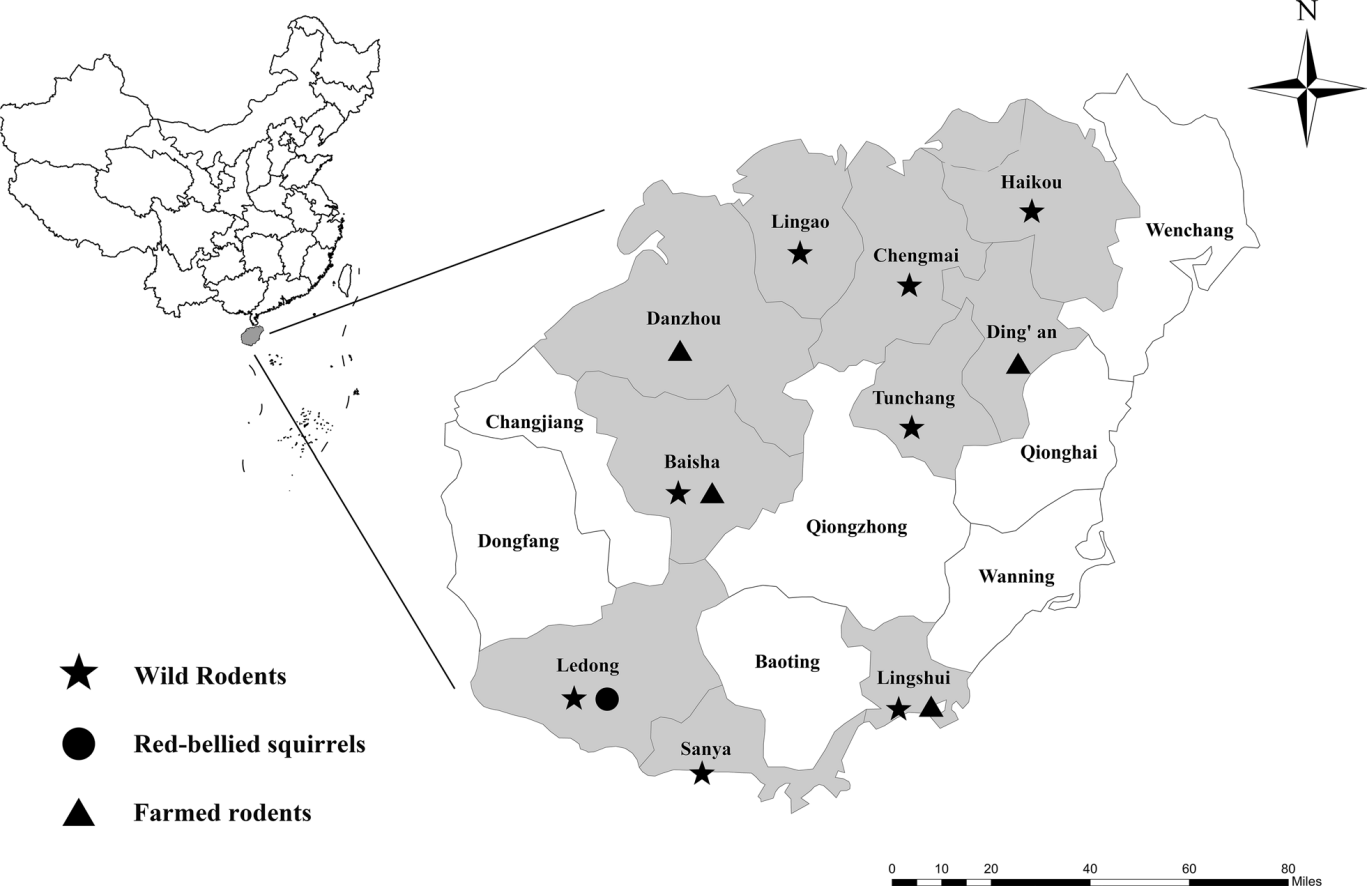
Specific locations where samples were collected in this study

A total of 603 fecal samples were collected from 210 farmed rats, 369 wild rats, and 24 red-bellied squirrels from ten cities in Hainan, China, between 1 December 2017 and 31 October 2019 (Fig. 1, Table 2). For capturing the wild rats, 20 cage traps were installed at each location containing peanut/sesame butter and sunflower seeds as the bait. The cages were positioned at sunset 5 m apart in transects and collected before sunrise. Within 48 h of capture, the wild rats were euthanized through CO_2_ inhalation, followed by the collection of fresh fecal specimens (~500 mg) from the intestines.

**Table 2:** Prevalence and distribution of *E. bieneusi* genotypes in wild and farmed rodents in the Hainan Province, China

Rodent species	No. of specimens	*E. bieneusi*	
		No. positive (%)	Genotype (*n*)
Wild rats			
Asian house rat (*Rattus tanezumi*)	134	31 (23.1)	PigEbITS7 (16); D (12); ESH-02 (1); Type-IV (1); EbpA (1)
Brown rat (*Rattus norvegicus*)	56	8 (14.3)	D (3); PigEbITS7 (1); Type IV (1); Peru 8 (1); HNR-I (1); HNR-II (1)
Edwardʼs long-tailed rat (*Leopoldamys edwardsi*)	38	3 (7.9)	D (2); HNR-III (1)
Chestnut white-bellied rat (*Niviventer fulvescens*)	10	0 (0)	na
Chinese white-bellied rat (*Niviventer confucianus*)	33	6 (18.2)	D (3); PigEBITS7 (2); Type-IV (1)
Indo-Chinese forest rat (*Rattus andamanensis*)	54	5 (9.3)	D (3); Type-IV (1); HNR-III (1)
Lesser rice-field rat (*Rattus losea*)	44	16 (36.4)	HNR-VII (15); D (1)
Subtotal	369	69 (18.7)	D (24); PigEbITS7 (19); HNR-VII (15); Type IV (4); HNR-III (2); Peru 8 (1); EbpA (1); ESH-02 (1); HNR-I (1); HNR-II (1)
Wild squirrels			
Red-bellied squirrel (*Callosciueus erythraeus*)	24	1 (4.2)	D (1)
Farmed rodents			
Asiatic brush-tailed porcupine (*Atherurus macrourus*)	93	7 (7.5)	D (3); HNR-VI (2); S7 (1); CHG5 (1)
Bamboo rat (*Rhizomyidae*)	117	18 (15.4)	D (15); Peru 11 (1); HNR-IV (1); HNR-V(1)
Subtotal	210	25 (11.9)	D (18); HNR-VI (2); S7 (1); CHG5 (1); Peru 11 (1); HNR-IV (1); HNR-V(1)
Total	603	95 (15.8)	D (42); PigEbITS7 (19); HNR-VII (15); Type IV (5); HNR-III (2); HNR-VI (2); EbpA (1); Peru 8 (1); Peru 11 (1); ESH-02 (1); S7 (1); CHG5 (1); HNR-I (1); HNR-II (1); HNR-IV (1); HNR-V (1)

*Abbreviation*: n, number of specimens; na, not applicable

For the farmed rats, a total of 117 bamboo rats and 93 Asiatic brush-tailed porcupine feces samples were collected from tanks each housing 1–3 animals. A single fecal sample (~15 g) was gathered from each tank (using disposable gloves), and approximately 30% of the total animals of each farms were collected, to minimize duplicate sampling.

Additionally, 24 fecal samples were collected from red-bellied squirrels which were captured from the Jianfeng Ling (18°23′–18°52′N, 108°44′–109°02′E).

### DNA extraction

Each fecal sample was centrifuged at 1500×*g* for 10 min at room temperature, followed by isolation of genomic DNA from each sample (~ 200 mg) using the QIAamp DNA Mini Stool Kit (Qiagen, Hilden, Germany) following the manufacturer’s instructions. The lysis temperature was raised to 95 °C to obtain a higher yield. AE elution buffer (200 ml) was used to elute the DNA, followed by storage at − 20 °C for further PCR analysis.

### Identification of wild rat species

A 421-bp sequence of the cytochrome *b* (*cytb*) gene in the fecal DNA was amplified using PCR to identify the rat species following a previously identified method [28]. The PCR cycle had the following parameters: 94 °C for 5 min, 35 cycles at 94 °C for 30 s, 51 °C for 30 s, 72 °C for 30 s, and a final step at 72 °C for 5 min.

### Genotyping of *E. bieneusi*

Nested PCR amplification of the ITS region was carried out to identify and genotype *E. bieneusi* using TaKaRa TaqDNA Polymerase (TaKaRa Bio Inc., Tokyo, Japan) along with genotype BEB6 DNA from deer (positive control) and 2 μl distilled water (negative control) using the primers and cycle parameters of Buckholt et al. [29]. The PCR products were analyzed using 1.5% agarose gel electrophoresis, followed by visualization by DNAGREEN staining (Tiandz, Inc., Beijing, China).

### DNA sequencing and analysis

The PCR products that were *E. bieneusi*-positive underwent bidirectional sequencing (Sangon Biotech Co., Ltd., Shanghai, China). Further PCR products were sequenced as necessary. Basic Local Alignment Search Tool (BLAST) and ClustalX 1.83 were used for genotyping of the *E. bieneusi* isolates by comparing the identified nucleotide sequence with the sequences published on GenBank. The genotypes were labeled following established nomenclature based on the 243-bp ITS region of *E. bieneusi*.

### Phylogenetic analysis

A neighboring-joining phylogenetic tree was constructed using Mega X software with the Kimura-2-parameter model and with 1000 replicates to evaluate the relationship between the genotypes identified in this study and to confirm the gene group.

### Nucleotide sequence accession numbers

The newly generated sequences were deposited in the GenBank database under the accession numbers MN267052-MN267057 and MN931659.

## Results

### Identification of the rat species

PCR sequencing of the *cytb* gene showed that the 369 samples of wild rats comprised 38 Edward’s long tailed rats (*Leopoldamys edwardsi*), 44 lesser rice-field rat (*R. losea*), 134 Asian house rats (*R. tanezumi*), 10 chestnut white-bellied rats (*Niviventer fulvescens*), 56 brown rats (*R. norveqicus*), 54 Indo-Chinese forest rats (*R. andamanensis*) and 33 Chinese white-bellied rats (*N. confucianus*). All *cytb* sequences showed 99–100% similarity to the following GenBank reference sequences: MG748345 for *R. tanezumi*; MG748255 for *N. fulvescens*; KT808632 for *R. norveqicus*; MG748260 for *R. andamanensis*; MG748257 for *R. losea*; KP992477 for *L. edwardsi*; and JF714942 for *N. confucianus.*

### Prevalence of *E. bieneusi*

We detected *E. bieneusi* in 15.8% (95/603) of the rodent samples, including 18.7% (69/369) of the wild rats, 4.2% (1/24) of the red-bellied squirrels, and 11.9% (25/210) of the farmed rodents (Table 2). Among the wild rats, the lesser rice-field rats had the highest prevalence of *E. bieneusi* (16/44, 36.4%), followed by Asian house rats (31/134, 23.1%), Chinese white-bellied rats (6/33, 18.2%), brown rats (8/56, 14.3%), Indo-Chinese forest rats (5/54, 9.3%) and Edward’s long-tailed rats (3/38, 7.9%). None of the 10 chestnut white-bellied rats were infected with *E. bieneusi*. Among the farmed rodents, the rate of prevalence of *E. bieneusi* in bamboo rats (18/117, 15.4%) was higher than that of the Asiatic brush-tailed porcupines (7/93, 7.5%). Only one of the 24 (4.2%) red-bellied squirrels was infected with *E. bieneusi*.

### Characterization and distribution of the genotypes of *E. bieneusi*

We identified 16 genotypes containing 41 polymorphic sites, including 9 known genotypes (D, Type IV, PigEBITS7, Peru8, Peru11, ESH02, S7, EbpA and CHG5) and 7 novel genotypes (HNR-I to HNR-VII (GenBank: MN267052-MN267057 and MN267057)) based on the ITS sequencing of the 95 *E. bieneusi* isolates (data not shown). Amongst them, genotype D (44.2%, 42/95) predominated, followed by PigEBITS7 (20.0%, 19/95), HNR-VII (15.8%, 15/95), Type IV (5.3%, 5/95), HNR-III (2.1%, 2/95), HNR-VI (2.1%, 2/95) and each of the remaining 10 genotypes (1.1%, 1/95) (Table 2).

Nucleotide sequence analysis showed that the novel genotypes HNR-I, HNR-II, HNR-VI and HNR-VII had the largest similarity with genotypes Peru8 (GenBank: MF476880), Type IV (GenBank: KP994661) SCR06 (GenBank: MK909573) and S (GenBank: AY945809) with one base insert at position 10 (a single nucleotide “A” insertion), 244 (G→A), 222 (C→A) and 134 (T→C), respectively. Genotype HNRM-III had two base differences at positions 118 (G→T) and 144 (A→G) compared to genotype HLJ-I (GenBank: KJ475402) from pigs in Heilonjiang, China. In contrast, genotypes HNR-IV and HNR-V had the largest similarity with genotypes YNM1 (GenBank: MG999511) and D (GenBank: KU557672), with six and five base differences at positions 3 (A→G), 65 (G→T), 226 (G→A), 234 (G→A), 235 (G→T) and 243 (G→T), and 226 (G→A), 234 (G→A), 235 (G→T), 237 (G→A) and 243 (G→T), respectively.

We observed a varied distribution pattern of the *E. bieneusi* genotypes among different rodent species (Table 2). Genotype D was found in all rodent species which were positive for the pathogen. Genotypes PigEbITS7, Type IV, Peru 8, EbpA, ESH-02, HNR-I to HNR-III and HNR-VII were found in wild rodents with genotype PigEbITS7 and Type IV in Asian house rats, Chinese white-bellied rats and brown rats; ESH-02 in Asian house rats; Peru 8, HNR-I and HNR-II in brown rats; HNR-III in Edward’s long-tailed rats and Indo-Chinese forest rats, genotype HNR-VII in lesser rice-field rats; EbpA in Asian house rat. On the contrary, genotypes S7, CHG5, Peru 11 and HNR-IV to HNR-VI were present in farmed rodents with genotypes HNR-VI, S7, and CHG5 in Asiatic brush-tailed porcupines and genotypes Peru 11, HNR-IV and HNR-V in bamboo rats (Table 2). Genotype D was detected in the *E. bieneusi* isolate from the red-bellied squirrel.

### Phylogenetic analysis

**Fig. 2:**
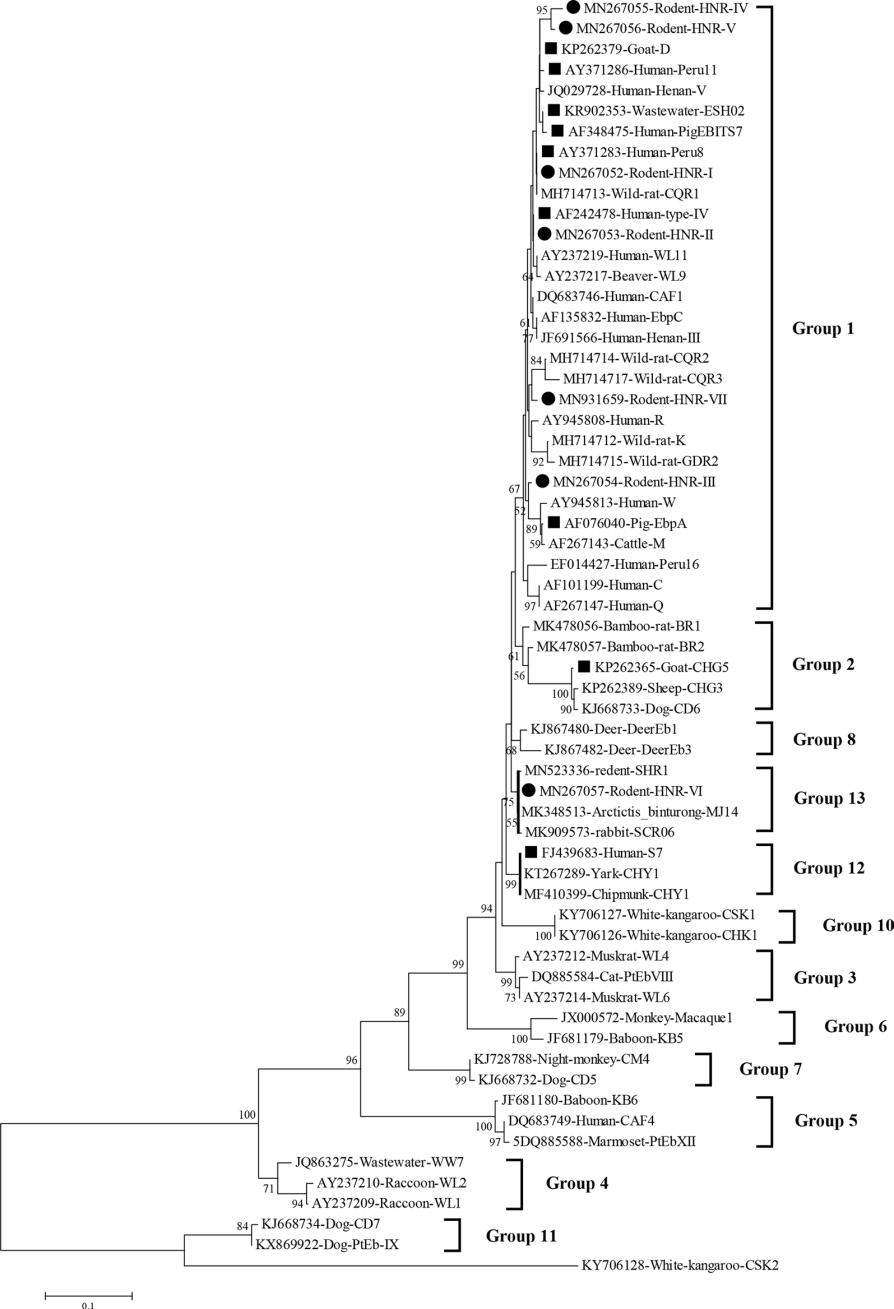
Phylogenetic relationships of *E. bieneusi* genotypes identified in the present study and other known genotypes deposited on GenBank was inferred by a neighboring-joining phylogenetic analysis of ITS sequences using the Kimura-2-parameter model and with 1000 replicates. Each sequence is identified by its accession number, host origin, and genotype designation. The *E. bieneusi* genotype CSK2 (GenBank: KY706128) from the white kangaroo was used as the outgroup. The black circles and squares indicate the known and novel genotypes identified in this study, respectively

The phylogenetic analysis of the ITS region of *E. bieneusi* divided the identified genotypes into the following four groups: Group 1 (*n* = 13); Group 2 (*n* = 1); Group 12 (*n* = 1); and the novel Group 13 (*n* = 1) (Fig. 2).

## Discussion

To the best of our knowledge, this study is the first report on the identification of *E. bieneusi* in rodents in Hainan, the southernmost province of China. Currently, there are 14 studies reporting the presence of *E. bieneusi* in rodents from six different countries (Table 1) [11–25]. These studies describe the prevalence of *E. bieneusi* infection among the rodents in the range of 1.1–100.0% (Table 1) [11–25]. Geographical location-based variation in the overall prevalence of *E. bieneusi* in rodents has been reported: 87.55% (7/8) in Peru [22]; 38.9% (121/311) in Poland [23]; 35.9% (52/145) in the USA [11, 25]; 12.2% (278/2272) in China [12–20]; 10.7% (31/289) at the Czech Republic and Germany border [21]; and 1.1% (3/280) in Slovakia [24]. These studies also reported species-based variation in the prevalence of *E. bieneusi* infection: 87.5% for guinea-pigs; 48.3% for prairie dogs; 39.1% for voles; 24.3% for hamsters; 5.1% for bamboo rats; 16.7–42.9% for squirrels; 4.0–7.9% for rats; 3.6–71.4% for chipmunks; and 1.1–87.5% for mice (Table 1) [12–25]. Notably, except for China and the USA, only one study was performed in each of the other countries and thus further large-scale surveillance studies should be conducted to ascertain these findings. We also found a variation in the prevalence of *E. bieneusi* infection in the rodents studied here. Thus, the prevalence of *E. bieneusi* was 16.6% in the wild rats, 4.2% in the red-bellied squirrels, and 11.9% in the farmed rodents. Wild rats showed a significantly higher rate of *E. bieneusi* infection compared with farmed rats and squirrels.

Among the 16 identified *E. bieneusi* genotypes, genotypes D, Peru8, PigEbITS7, Type IV, Peru11 and EbpA are known human pathogens [5]. Genotype D was the predominant genotype which was found in 44.2% (42/95) of *E. bieneusi* isolates. This genotype was widely distributed and present in all sampled rodent species. It is also commonly found in human infections in more than 20 countries and has been isolated from more than 25 animal species and water samples [5]. Genotype PigEbITS7 was found in Asian house rats, brown rats and Chinese white-bellied rats. This genotype was originally identified in pigs in Massachusetts, USA, and in immunocompromised patients in Ahvaz in Iran, and Gangxi and Henan, China [29–32]. In addition to pigs and humans, genotype PigEbITS7 has been identified in monkeys and bamboo rats from China [9, 12]. Genotypes Peru8, type IV and Peru11 were detected in a single rat species but are known to be human and animal pathogens [5]. Thus, the identification of the above mentioned six genotypes in rodents indicated transmission of parasites from infected rodents to humans as well as other animals.

The remaining two known genotypes, ESH-02 and S7, were found in Asian house rats and Asiatic brush-tailed porcupines, respectively. Genotype ESH-02 (also named Ind 1) was originally identified in wastewater treatment plant effluents in Shanghai, China [33], and also in renal transplant recipients and AIDS patients in India [34]. There are no published reports of the presence of this genotype in any animal species. This study confirmed for the first time that genotype ESH-02 can infect rats, suggesting its zoonotic potential. Genotype S7 (also named CHY1) was previously identified in an immunosuppressed patient in the Netherlands in 2009 [35], yaks in Henan, China [36], chipmunks and rabbits in Sichuan, China [17] and experimental rats in Henan, China [15]. We found that this genotype of *E. bieneusi* was also found in Asiatic brush-tailed porcupines. These finding indicated that genotype S7 has a wide range of animal reservoirs and potential for zoonotic transmission. Further studies should be conducted to explore additional animal reservoirs of these genotypes.

In this study, 13/16 (81.3%) genotypes and 95.8% (91/95) of the *E. bieneusi* isolates belonged to Group 1. The genotypes in this group has been identified in several hosts, such as humans, and possess a high potential for cross-species and zoonotic transmission of *E. bieneusi* [5]. Group 1 was deemed zoonotic based on the prevalence of genotypes such as Type IV, D, Peru11, EbpC, and Peru8 in several animal hosts [5]. The fact above suggesting that the *E. bieneusi*-infected rodents posed a serious threat to the local inhabitants. Meanwhile, the identification of genotype HNR-VII belonging to the novel Group 13, was a unique epidemiological feature of *E. bieneusi* in rodents in Hainan Province of China.

## Conclusions

Our novel data demonstrate a high rate of prevalence of *E. bieneusi* infection in various rodent species in Hainan, China. The finding of zoonotic *E. bieneusi* genotypes (PigEbITS7, Peru8, D, Type IV, Peru11, EbpA, S7 and ESH-02) in rodents suggests that they may pose a serious public health threat in the area. Moreover, the seven novel genotypes provided novel insights into the genotypic variations of *E. bieneusi.* Adequate control of rodents and public education on the management of rodent feces should be implemented in these areas.

## Data Availability

All data generated or analysed during this study are included in this published article. Sequences were submitted to the GenBank database under the accession numbers MN267052-MN267057 and MN931659.

## Abbreviations

AIDS: acquired immunodeficiency syndrome
BLAST: basic local alignment search tool
ITS: internal transcribed spacer
